# Economic development and wage inequality: A complex system analysis

**DOI:** 10.1371/journal.pone.0182774

**Published:** 2017-09-19

**Authors:** Angelica Sbardella, Emanuele Pugliese, Luciano Pietronero

**Affiliations:** 1 ISC-CNR – Institute of Complex Systems, Rome, Italy; 2 Department of Physics, Sapienza Università di Roma, Rome, Italy; 3 Faculty of Economics, Università degli Studi di Roma Tor Vergata, Rome, Italy; University of Warwick, UNITED KINGDOM

## Abstract

Adapting methods from complex system analysis, this paper analyzes the features of the complex relationship between wage inequality and the development and industrialization of a country. Development is understood as a combination of a monetary index, GDP per capita, and a recently introduced measure of a country’s economic complexity: Fitness. Initially the paper looks at wage inequality on a global scale, over the time period 1990–2008. Our empirical results show that globally the movement of wage inequality along with the ongoing industrialization of countries has followed a longitudinally persistent pattern comparable to the one theorized by Kuznets in the fifties: countries with an average level of development suffer the highest levels of wage inequality. Next, the study narrows its focus on wage inequality within the United States. By using data on wages and employment in the approximately 3100 US counties over the time interval 1990–2014, it generalizes the Fitness-Complexity metric for geographic units and industrial sectors, and then investigates wage inequality between NAICS industries. The empirical time and scale dependencies are consistent with a relation between wage inequality and development driven by institutional factors comparing countries, and by change in the structural compositions of sectors in a homogeneous institutional environment, such as the counties of the United States.

## Introduction

The upswing in inequality in industrialized Western societies in the last decades of the 20th century renewed the debate on the distribution of income and wealth, a central issue in economic and political debates since the birth of economics as a discipline [1, 2]. In this paper our aim is to investigate the co-evolution of economic development with the inequality of labor income in a complex network framework. In particular, we will look at the empirical evidence to check if it is consistent with the narrative of the relationship between wage inequality and development mostly driven by two major drivers: changes along the development path of i) institutions and ii) the sectoral composition of the labor force. Our main contribution will be the attempt to properly identify the objects of our research. Indeed, many definitions of development, growth and inequality have been proposed. Since many terms are broadly defined, it is difficult to empirically verify theoretical models. Identifying and defining *development* and *inequality* also means finding suitable metrics to assess them quantitatively.

Indeed, development and growth are often confused with an increase in the size of a economy—usually measured as GDP percentage increases. Quoting Nelson: “Contemporary formal growth theories treat economic growth as almost all quantitative. On the other hand, in the historical accounts, lots of qualitative things are happening. New technologies are emerging, and so also are new forms of business organization, and new institutions. Put another way, […] Development is moving forwards and not simply things getting bigger or smaller or staying the same size” [3]. The development of a country is a complex and dynamic phenomenon, and its analysis requires a detailed understanding of historical peculiarities, relations between endogenous and exogenous factors, technological change and capacity of innovation, firms’ characteristics, institutional behaviors and capital investment paths. All of these are the intangible endowments of capabilities that characterize an economy and may lead to very different evolution paths. Therefore, the view that purely monetary GDP measures cannot account for national progress and development is far from new. Over recent years, alternative or complementary indicators have been introduced [4–6].

In the Economic Complexity literature there is a vivid debate on how the notion of complexity might integrate with the information given by monetary indexes [7, 8]. In a recent strand of literature Tacchella et al., by employing time series on the export network of countries, introduced Fitness, a new dimension to assess the complexity of a country and to indirectly capture through an empirical algorithm its unobservable manufacturing capabilities [8–10]. In this framework, for example, Hartmann et al. [11] by looking at the relationship between income inequality, institutions and economic complexity find a decreasing relationship between the complexity of a country—measured by the Economic Complexity Index [7] and other indexes—and income inequality. In the present work we will follow the approach of Pugliese et al. [12] who define a relative development metric as a combination of a monetary value, relative GDP per capita, and one of the above non-monetary measures of the complexity of a country’s productive system, Fitness. We will firstly generalize the metric developed by Tacchella et al. [8] for more general networks. To do so, we will employ United States’ data on wages and employment levels grouped by economic sectors at a county level; we will compute county Fitness by looking at the localization of industrial sectors in each county, as is common in geographical economics [13]. Then, by combining the rankings of GDP per capita and Fitness, we will use an approach similar to the one used by Pugliese et al. [12] to define a development measure. We will call this the Complex Relative Rank Development index. By identifying development not merely through using monetary statistics but also by conveying information on capabilities through Fitness, we will be able to better characterize the relationship between wage inequality and development.

With regards to inequality, we will focus on wage inequality. This is because wages constitute a major component of total income, and the rise of wage inequality in the last decades has notably influenced the movement of overall income inequality [14–16]. Furthermore wages—which reflect, among other factors, the skill levels of workers—are directly related to industrial development, and so to innovation, technological change and structural dynamics [17, 18]. The inequality of a distribution, i.e. the dispersion within it, can be measured in several ways, depending on which features of inequality are more significant to the analysis: the Gini coefficient, the Coefficient of Variation, the Theil index, the Herfindhal index, the ratio between the top 10% and the bottom 10% and so on, each of which represents a different quantitative aspect of what we simply call inequality. Here, since we employ labor income and employment data grouped by industrial sector, to quantify wage inequality we use a Theil measure between groups, similarly to the *University of Texas Inequality Project* [19–21]. The Theil index, being an entropy-based measure, is naturally decomposable among population groups and therefore it allows us to study a system and its components in a consistent way. However, we will show that our findings are broadly unaltered if, for example, we use a Gini index (see the S1 Appendix in the Supporting Information).

Which models have been proposed to describe the relation between wage inequality and the process of development? One of the most influential arguments was made by Simon Kuznets in 1955 [22], who hypothesized that in the first phase of industrialization inequality would rise and then, as industrialization entered a further stage, it would decrease, tracing the renowned inverted U-shaped “Kuznets curve”. In fact, when the passage towards industrialization takes hold there is an injection of cheap rural labor to the cities that holds down wages. This provokes a wide urban-rural gap and a consequent rise of inequality, thus creating a relationship between development and wage inequality in which the *inter-sectoral transition* from agriculture to industry has a primary role. Kuznets’ innovation is the way in which he relates economics and institutions. He predicts that, when capitalism enters its advanced phase, the labor force bargains to improve pay and work conditions through social struggle, ultimately leading to a strengthening of the welfare state, and a process of democratization is triggered in which modern industrial relations take place and inequality decreases. All of these are elements that pave the way for social democracies, in which pay inequality is supposed to decrease almost mechanically: the second phase of industrialization is led by *institutions*. The full Kuznets curve, the increase and decrease of inequality while the country develops, is therefore postulated through a mix of sector shifts and institutional changes. To disentangle these two forces, we will look at this process on two different scales: among countries and inside one country, the United States. By looking at the relationship between wage inequality and development at different scales we are able to reconstruct evidences that are broadly compatible with Kuznets’ theory.

Of course, various and often contrasting views about Kuznets’ hypothesis have been expressed, both empirically and theoretically, from very different starting points. Empirically, relating different measures of inequality to different possible definitions and measures of development, divergent results emerge. This spans from the recovery of the entire curve [23, 24] or only its downward sloping half [14, 25–27], to a parabolic or upward sloping curve [28–31], which advocates a resurgence of inequality especially in the industrialized world [32, 33]. Or even to the conclusion that no systematic pattern in the movement of inequality in the course of development exists [34]. Theoretically a Kuznets’ process on the one hand is formalized and described as an “economic law” [35–37], while on the other hand many scholars question the egalitarianism of developed societies, often reversing the lines of causality. The recent polarization of wages in wealthy countries, for example, is seen as a direct consequence of the deindustrialization of late capitalist development and to the consequent “skill-biased technical change” [38–44]. Even the opposite causal relationship, the role of inequality in explaining the development of a country, is considered: through increased political and economic inclusivity as the engine for development and growth [45–47] or, conversely, through increased inequality as a prerequisite for accelerated growth [18, 48, 49]. Or, for instance from a demand side perspective, “Big Push” models predict the role of a sizable middle class in producing the necessary demand to trigger industrialization [50, 51]. The causal relationships are multifaceted and likely to go in multiple directions, implying an underlying complex process, that requires the correct analytical tools to be analyzed.

The remainder of this paper is structured as follows. In the next section, *Materials and methods*, we will first describe the sources of data that we use, and later will introduce our variables of interest: Fitness as a measure of economic complexity, the Complex Relative Rank Development index as a measure of development, and a Theil index for the inequality among industrial sectors. Section *Results* will show our results and propose a consistent narrative. Section *Conclusion* will summarize the argument and offer the concluding observations.

## Materials and methods

### Sources of data

In the first part of the paper we will focus on a pooled analysis of a panel of countries. While, in the second part, we will narrow the field of our investigation and we will examine cross-sections of the US counties. In the followings we briefly illustrate the employed data.

#### World Table 8.1 (PWT)

The GDP and population of countries are taken from the Penn World Table 8.1 (PWT). PWT is a database produced by the *University of Groningen* and the *University of Pennsylvania* and it provides levels of income, output, input and productivity, covering 167 countries over the period 1950–2011 [52]. In particular, we employ data on Expenditure-Side Real GDP at current purchasing power parities and population levels.

#### Export Fitness

In the multi-country analysis, as a measure of the Economic Complexity of countries we take their Export Fitness, a dimension recently introduced in Tacchella et al. [8]. For a more detailed discussion of Fitness we refer to the Subsection *Fitness as a measure of Economic Complexity*. The Export Fitness data-set covers a number of countries varying slightly between 145 and 148, over the period 1995–2010. The export volumes considered to evaluate the Fitness comprise 1131 products and are taken from the BACI database [53] in which the exported commodities are classified according to the 4-digit Standard International Trade Classification (SITC), Revision 2.

#### University of Texas Inequality Project (UTIP-UNIDO)

In the multi-country analysis, we employ the measure of wage inequality produced by the *University of Texas Inequality Project* (UTIP) with the support of *INET* in the UTIP-UNIDO data-set [54]. The latter is a global data-set which encompasses a Theil measure of pay inequality across manufacturing sectors covering 167 countries over the period 1963–2008. The data on wages from which the index is built is drawn from the Industrial Statistics database published annually by the *United Nation Industrial Development Organization* (UNIDO), where industrial sectors are categorized according to the International Standard Industrial Classification (ISIC) at a 2 or 3-digit aggregation level.

#### Bureau of Labor Statistics (BLS)

The data on employment and wages regarding the United States is taken from the Quarterly Census of Employment and Wages data-set (QCEW) of the US *Bureau of Labor Statistics* over the period 1990–2014 [55]. In the QCEW industries are labeled by the North American Industry Classification System (NAICS), a standard method used in Canada, Mexico, and the United States to classify business establishments according to types of economic activity. The NAICS numbering system employs a 6-digit code at the most detailed industry level. For the approximately 3100 US counties or county equivalents, the QCEW provides employment and earnings information at a county-level by NAICS industry and by ownership sector. This data is also aggregated to annual levels, to higher industry levels and to higher geographical levels (national, state). In our analysis we look at the private sector on a county-level, and we aggregate the data at 6-digit NAICS into wages and employment levels at 3-digits.

### Variables of interest

#### Fitness as a measure of Economic Complexity

As mentioned in the Introduction, in a recent strand of literature Tacchella et al. defined Fitness, a new measure for the economic complexity of a country [8–10, 56] inspired by previous works on Economic Complexity [7]. Fitness is an indirect measure of the manufacturing capabilities of a country. The capabilities are representative of the underlying social and economic structure of a society, and are the sum of all those national characteristics that enable a country to produce and export goods. By describing the international goods market as a bipartite network of countries and products, the measure defined by Tacchella et. al. provides a ranking of the development potential of countries quantifying the diversification and the complexity of their export baskets. They created a non-linear coupled map the fixed point of which defines a metric for the Fitness of countries and the Complexity of their products. From now on we will refer to this map as the Fitness-Complexity algorithm. Given that the Fitness-Complexity algorithm defines an intensive metric, it is possible to generalize its definition, taking into consideration a bipartite network of regions and economic activities—in which by region we mean a generic geographical unit that can be a sub-division of the same country or a macro-area. By doing so, it would be possible to give information on the whole economy of a country, not only on its manufacturing capabilities.

In order to generalize in such a way the Fitness-Complexity algorithm, in the analysis within the United States, rather than focusing exclusively on export, we consider its industrial production as a whole. Instead of export data we employ labor data, the counties of the United States as geographical units and 3-digit NAICS sectors as economic activities. Data on wages and employment is taken from the Quarterly Census of Employment and Wages data-set of the *Bureau of Labor Statistics* described in Subsection *Sources of data*. In a work soon to be submitted, we are exploring in depth this generalization of the algorithm for the United States and its application on different geographical scales (i.e. states and counties) and different industrial aggregations.

To formally express this idea, as a reasonable indicator of productive system “dimensions”, we employ the total earnings *W*_*rs*_ of the workers of sector *s* in region *r* over one year. Thus, if we take *N*_*s*_ economic sectors and *N*_*r*_ regions, we can build a region-sector matrix $\hat{M}$ of dimension *N*_*r*_ × *N*_*s*_. Initially, the matrix element *M*_*rs*_ reports the dimensions of the system, with the entries being the wage volume *W*_*rs*_ for *r* = 1, ⋯, *N*_*r*_ and *s* = 1, ⋯, *N*_*s*_. Then, along the same lines of the Fitness-Complexity algorithm, in order to remove any trivial correlations with wage volumes, in matrix $\hat{M}$ we report only whether a sector is present or not in a region. When *s* is present in *r*, we assign to the elements *M*_*rs*_ of the matrix the value 1, and the value 0 otherwise. The digitization criterion used to make $\hat{M}$ binary is an index closely related to a classical tool of economic geography, the Location Quotient (LQ). *LQ*_*rs*_ of sector *s* for region *r* is a ratio that allows us to compare the distribution of employment by industrial sector in an area with a reference distribution, which in general is the national one. If *E*_*rs*_ is the number of workers in sector *s* for region *r*, the Location Quotient is expressed by the formula:

$$\begin{array}{c} LQ_{rs}=\frac{E_{rs}}{\sum_{r^{\prime }}E_{r^{\prime }s}}/\frac{\sum_{s^{\prime }}E_{rs^{\prime }}}{\sum_{r^{\prime }s^{\prime }}E_{r^{\prime }s^{\prime }}}. \end{array}$$ (1)

When *LQ*_*rs*_ = 1 the share of employment in sector *s* is equal for the regional and the national economy; while when *LQ*_*rs*_ < 1 the share of regional employment is less than it is in the national case; and vice versa for *LQ*_*rs*_ > 1.

By defining *w*_*rs*_ as the average wage of *s* in *r*, we construct a Location Quotient, that we will call the Wage Location Quotient (WLQ), which makes possible to compare distribution of wages, in formula:

$$\begin{array}{c} WLQ_{rs}=LQ_{rs}\cdot \frac{w_{rs}}{\sum_{r^{\prime }}w_{r^{\prime }s}}/\frac{\sum_{s^{\prime }}w_{rs^{\prime }}}{\sum_{r^{\prime }s^{\prime }}w_{r^{\prime }s^{\prime }}} \end{array}$$ (2)

which is equivalent of writing:

$$\begin{array}{c} WLQ_{rs}=\frac{W_{rs}}{\sum_{r^{\prime }}W_{r^{\prime }s}}/\frac{\sum_{s^{\prime }}W_{rs^{\prime }}}{\sum_{r^{\prime }s^{\prime }}W_{r^{\prime }s^{\prime }}}. \end{array}$$ (3)

*WLQ*_*rs*_ is the ratio of the wage share of sector *s* in region *r* to the wage share of sector *s* in the whole geographic area under consideration. We identify a region *r* as comparatively competitive in a particular industry *s* if *WLQ*_*rs*_ ≥ 1, i.e. if its share of wages in *s* is above the average. Note that the expression in Eq 3 is the direct counterpart of the Revealed Comparative Advantage [57] used in international trade.

Therefore we can digitize matrix $\hat{M}$ in the following manner: when *r* is competitive in *s*, we assign to the matrix element *M*_*rs*_ the value 1, and the value 0 otherwise. That is:

$$\begin{array}{c} M_{rs}=\{\begin{array}{cc} 1 & \text{if}\;WLQ_{rs}\geq 1\\ 0 & \text{otherwise.} \end{array} \end{array}$$ (4)

Thus, from matrix $\hat{M}$ it is possible to obtain an intensive metric that measures region Fitness *F*_*r*_ as the diversification weighted by the sector complexities, and sector Complexity *Q*_*s*_ as the diversification bounded by the Fitness of the less competitive region in which the sector is present. Between these two variables a non-linear coupling holds. This is expressed in the Fitness-Complexity iterative algorithm (defined in Eqs 5 and 6) in which, at every step, *F*_*r*_ and *Q*_*s*_ are evaluated and normalized:

$$\begin{array}{c} \left\{ \begin{array}{cc} {\tilde{F}}_{r}^{\left(n\right)}=\sum_{s}M_{rs}Q_{s}^{\left(n-1\right)} & \\ {\tilde{Q}}_{s}^{\left(n\right)}=\frac{1}{\sum_{r}M_{rs}\frac{1}{F_{r}^{\left(n\right)}}} \end{array}\{\begin{array}{cc} F_{r}^{\left(n\right)}=\frac{{\tilde{F}}_{r}^{\left(n\right)}}{<{\tilde{F}}_{r}^{\left(n\right)}>} & \\ Q_{s}^{\left(n\right)}=\frac{{\tilde{Q}}_{s}^{\left(n\right)}}{<{\tilde{Q}}_{s}^{\left(n\right)}>}. \end{array}\right. \end{array}$$ (5)

With initial condition:

$$\begin{array}{c} \sum_{s}Q_{s}^{\left(0\right)}=1\;\forall s. \end{array}$$ (6)

The iteration of the coupled equations leads to a fixed point which has been proved to be stable and non-dependent on initial conditions [8]. The fixed point defines a non-monetary metric which quantifies the Fitness of the region in analysis and the Complexity of its economic sectors. This generalized Fitness-Complexity metric takes into account the overall economy, also those sectors that only have a role in domestic markets, such as different kinds of non exportable services.

**Fig 1:**
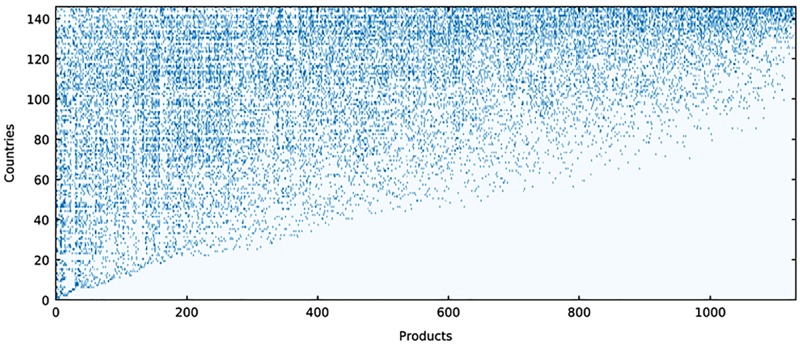
The binary matrix of countries and products built from the worldwide export flows of BACI data-set for 1998, with *N*_*countries*_ = 147 and *N*_*products*_ = 1131 [9]. Products are categorized according to the Harmonized System 2007 at 4-digit coarse-graining and the adopted digitization criterion is Balassa’s Revealed Comparative Advantage. By sorting the columns of the matrix by increasing Fitness and the rows by increasing Complexity, the matrix acquires a triangular-like shape. As it turns out, countries with more diversified export baskets are more competitive, while countries specialized in a few products—which generally are also exported by every other country—are the less competitive.

**Fig 2:**
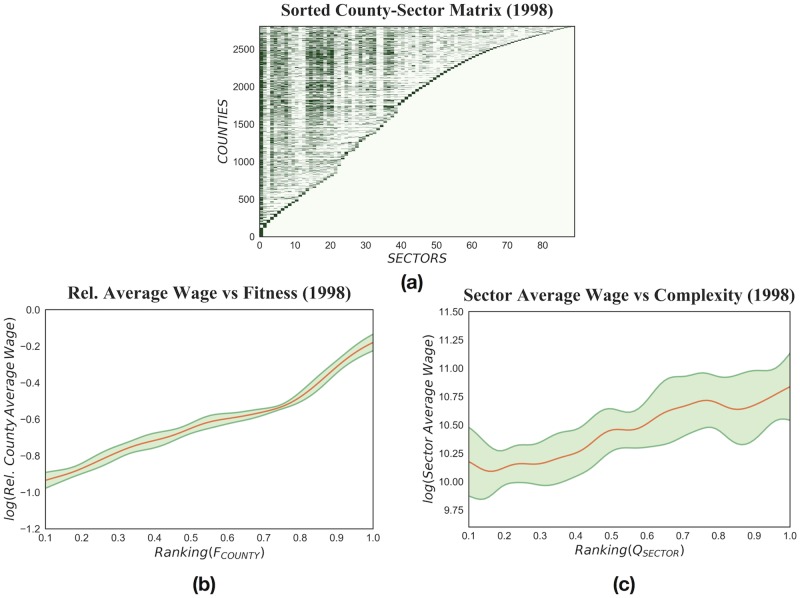
The three figures refer to the US counties in 1998 and are realized through considering data on wages and employment from the QCEW data-set. In this case, sectors are categorized according to the NAICS industry classification at 3-digit aggregation level, with *N*_*counties*_ = 2805 and *N*_*sectors*_ = 89. (a): The binary county-sector matrix $\hat{M}$ is built from the volume of sectoral wages. By ranking the columns of the matrix by increasing County Fitness and the rows by increasing Sector Complexity, $\hat{M}$ assumes a triangular-like shape, as it did in the analysis of international export. Hence, the path towards diversification in the production of goods and services is not only taken, as we have seen in Fig 1, by high Fitness countries but also by the most complex and diversified US counties. (b): The relation between the average wage and the Fitness of counties. The red line represents a kernel estimation of Relative County Average Wage versus *F*_*COUNTY*_ and the green shadowed area shows a 90% confidence interval of the expected value computed with bootstrap. On a country level the monetary counterpart of Fitness was the GDP per capita, while here we adopt the average aage as a proxy of the wealth produced by labor in a county. The trend is concordant with the one found comparing countries, in the sense that a non-linear relationship holds between the two variables: as *F*_*COUNTY*_ grows the Average Wage of the county increases; except for some deviations of Fitness from the monetary metric which are highly informative on a the potential performances of counties. (c): The relationship between national Sector Average Wage and Sector Complexity. As in the previous case, the red line is a kernel estimation of Sector Average Wage versus *Q*_*SECTOR*_ and the green area is a 90% confidence interval computed with bootstrap. As it seems reasonable, Sector Average Wage grows with Sector Complexity.

We use this metric in evaluating the performances of the industries of the United States at a county level over for the years 1990–2014. By looking at Figs 1 and 2, we can see that we recover the essential features of the metric based on international export. The county-sector matrix, once suitably sorted, shows triangular shape: the fittest counties are the most diversified—almost all sectors are present in their productive systems—while the regions specialized on few sectors are the less competitive (panel (a) of Fig 2); the Fitness and the Relative Average Wage of counties behave in a comparable way to GDP per capita and country Fitness (panel (b) of Fig 2); and in panel (c) of Fig 2 we notice that, as it seems sensible, the more complex is a sector the higher is its average wage. Given these results, the redefinition of the Fitness-Complexity algorithm presented in this section appears a solid and reliable instrument in studying the comparative development of US counties.

#### The Complex Index of Relative Development

One of the main novelty of this paper is the attempt to identify industrialization and development in a more nuanced and complete way; in order to do so, we take into consideration a recently introduced measure useful to study industrialization as a mono-dimensional process, the Complex Index of Relative Development (CIRD) [12], a linear combination of Fitness and GDP per capita. However, with respect to Pugliese et al. [12] and other works on the use of Fitness to forecast per capita GDP levels [56], here we use the rankings of GDP per capita and Fitness instead of their values. Thus our index, that we will call the Complex Index of Relative Ranking Development (CRRD), is defined as an arithmetic mean of Fitness ranking and GDP per capita ranking for region *r* at time *t*:

$$\begin{array}{c} CRRD_{r,t}=\frac{1}{2}(Ranking\left(F_{r,t}\right)+Ranking\left(GDPpc_{r,t}\right)). \end{array}$$ (7)

To ensure comparability in all sections, in this paper we always use the *CRRD* as an economic development measure; however, our results are robust to the use of *GDP* per capita and Fitness.

#### A Theil measure of the wage inequality among sectors

Analyzing the structure of the wage distribution by economic sector requires a suitable metric to determine its dispersion. Here, we adopt an *ad hoc* Theil index [19], a measure of inequality across income distributions defined by analogy with the Shannon Entropy [58]. The Theil index for a population of *n* individuals and for a discrete income distribution $y\in R_{+}^{n}$ is algebraically defined as:

$$\begin{array}{c} T=\frac{1}{n}\sum_{p=1}^{n}\frac{y_{p}}{\mu }\cdot \text{log}(\frac{y_{p}}{\mu }) \end{array}$$ (8)

where every *p* individual has income *y*_*p*_ for *p* = 1, …, *n* and $\mu =\bar{y}$ is the average income. The upper bond is dependent on the population size, *T* ∈ [0, *log*(*n*)]. When *T* = 0 there is perfect equality, the situation in which everyone has the same income *μ*. Instead, when *T* = *log*(*n*) inequality reaches its maximum and one individual owns all the income. Thus, by drawing this analogy with the Shannon Entropy, an inversion of extremal situations takes place: in fact, a state of maximum disorder corresponds to the minimum of the Theil index; on the contrary, when *T* is maximum there is minimum disorder.

One of the advantages, originally explored by Theil himself in 1967, of entropy-based inequality measures is that they are decomposable across population groups. This property proves to be very useful when analysing inequality in the wages among industrial sectors. We will explain why by following the approach and the formalism adopted by the *University of Texas Inequality Project* [59]. Indeed, if $Y=\sum_{p=1}^{n}y_{p}$ is the total income of the population, we can rewrite Eq 8 as it follows:

$$\begin{array}{c} T=\sum_{p=1}^{n}\frac{y_{p}}{Y}\cdot \text{log}\left(\frac{y_{p}}{Y}/\frac{1}{n}\right). \end{array}$$ (9)

As pointed out in Conceição and Ferreira [20], expressing *T* in the form of Eq 9: “highlights a possible intuitive interpretation of the Theil index as a direct measure of the discrepancy between the distribution of income and the distribution of individuals between mutually exclusive and completely exhaustive groups”. Grouping all the individuals in *m* groups—each group *i* (*i* = 1, …, *m*) having *n*_*i*_ individuals and total income *Y*_*i*_—we can parse overall inequality into a between-group and a within-group component [21, 59]:

$$\begin{array}{c} T=T_{g}^{\prime }+T_{g}^{w}. \end{array}$$ (10)

Where $T_{g}^{\prime }$ is the between-group component and it is given by:

$$\begin{array}{c} T_{g}^{\prime }=\sum_{i=1}^{m}\frac{Y_{i}}{Y}\text{log}\left(\frac{Y_{i}}{Y}/\frac{n_{i}}{n}\right). \end{array}$$ (11)

And $T_{g}^{w}$, the within-group component, is given by:

$$\begin{array}{c} T_{g}^{w}=\sum_{i=1}^{m}\frac{Y_{i}}{Y}T^{\left(i\right)} \end{array}$$ (12)

where *T*^(*i*)^ is the Theil index for each group *i* and accounts for the inequality between the members of group *i*. Several decomposition choices are possible, for instance one might concentrate on population characteristics—such as gender, age, race, economic sector of employment and so forth—or one might divide the population on the basis of their geographical residence.

In this paper, as in Galbraith and Hale [60], we will look at the dispersion of wages among industrial sectors; however in our case, the geographical units are counties. In general, over a certain period of time and in a specific geographical unit, we consider a partition of the working population in *N*_s_ mutually exclusive and completely exhaustive groups, with *N*_s_ being the number of economic sectors present in the society under study. If *P* is the total number of workers in the considered area and *μ* the average wage of all jobs, *y*_*i*_ the average wage of the *i*-th industrial sector and *p*_*i*_ the number of workers in sector *i*, then the between-sector wage inequality can be measured by a $T_{g}^{\prime }$ of the form:

$$\begin{array}{c} T_{sectors}^{\prime }=\sum_{i=1}^{N_{s}}\frac{p_{i}}{P}\frac{y_{i}}{\mu }\log(\frac{y_{i}}{\mu }). \end{array}$$ (13)

$T_{sectors}^{\prime }$ decreases when average wages in low-paying sectors rise, when they fall in high-paying sectors, or when sectors that are far from the overall average in either direction lose employment, and it increases in the opposite cases. $T_{sectors}^{\prime }$ is a group-based measure of the dispersion in the distribution of wages. It does not describe differences between individual workers, but it measures the inequality that results from the difference in average labor income between economic sectors and it is not sensitive to within-sector wage variability. Nevertheless, thanks to the decomposability properties of Theil measures, it has been shown that, under some formal criteria, a between-group Theil statistic also captures major characteristics of the evolution of pay inequality within industrial sectors and tracks the general movement of overall inequality in household incomes [59, 60].

Here, at a country-level, we employ the Theil measure developed by the *University of Texas Inequality Project*, while at a county-level we compute a between-sector Theil component for US counties.

Notice however that, while we chose the Theil index for its decomposition characteristics, Cowell et al. show that no inequality measures can be perfectly decomposable without being highly affected by single outlying observations [61]. To check for the eventuality that our results are driven by few spurious observations, in S1 Appendix we will replicate our exercise using the Gini index, a measure particularly stable to single outliers [61]. The trends found with the Gini index for evaluating wage inequality in the US counties are comparable to the one obtained with the Theil index which we then consider a satisfactory measure of between-sector inequality.

## Results

With the aim of exploring the relation that links the inequality in the distribution of labor income to economic development, we carry out an analysis *à la* Kuznets. As already mentioned, one of the main goals of our paper is the identification of development by considering Fitness as complementary to a more classic measure of economic performances, GDP per capita.

This section is organized as follows. In Subsection *A global analysis: wage inequality among countries* we will carry out a pooled cross-sectional study of development levels among countries. By using the *CRRD* defined by Eq 7 as a measure of development, we show that the relationship between development and wage inequality follows a curve that call to mind the one predicted by Kuznets. Next, in Subsection *Within one country: the case of the United States* we will focus on the same relation within the United States with the purpose of checking if the movement of wage inequality with development at this scale shows different features. In Subsection *Behind the curve* we will look for the potential drivers of the relation between development and wage inequality at different scales. In conclusion, in Subsection *Time evolution* we will analyze the time evolution of this relationship both at a country-level and within the United States.

### A global analysis: Wage inequality among countries

In this first exercise our purpose is to better tackle the relation to wage inequality and development when integrating the monetary information carried by GDP with that on country capabilities conveyed by Fitness. Thus, we expect to find a relation that might remember the Kuznets curve.

In practice, we will study with a cross-sectional approach the comparative development of countries proxied by the *CRRD* and wage inequality measured by the UTIP-UNIDO coefficient introduced in Subsection *Sources of data*. We will first explore these relationships through a continuous non parametric description [62] and, in order to examine time and space dimensions simultaneously, we will pool all countries and years for a number of countries varying between 145 and 148 and over the period 1990–2008. Later we will move to a more traditional parametric description to provide a quantitative counterpart to the qualitative results found.

**Fig 3:**
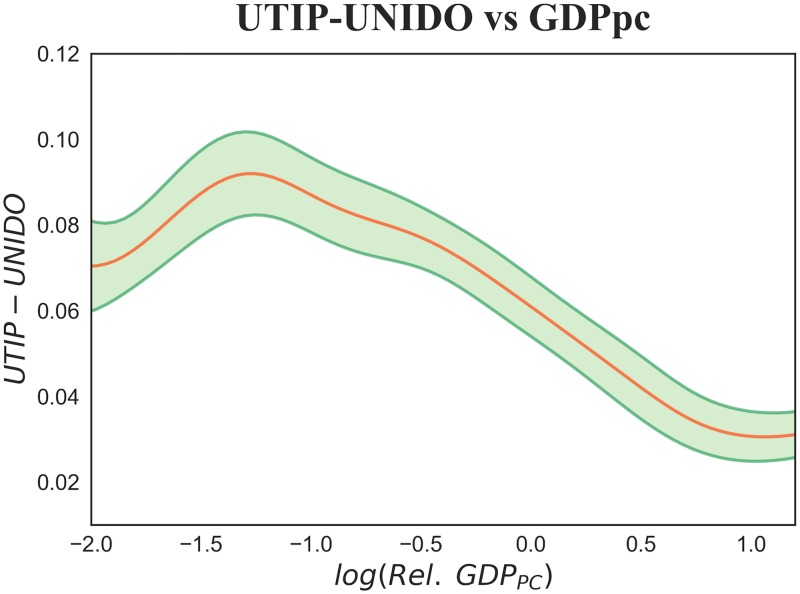
Pooling of all countries and years for a total of 936 observations, over the time interval 1990–2008 and for a number of countries varying between 145 and 148. The red line shows a non-parametric kernel estimation of the UTIP-UNIDO coefficient versus Relative GDP per capita. The green shadowed area represents a 90% confidence interval of UTIP-UNIDO expected values and has been computed with bootstrap. The negative relation reflects the one foreseen by the second half of the Kuznets curve: industrially advanced economies with high GDP per capita have low UTIP-UNIDO and vice versa.

**Fig 4:**
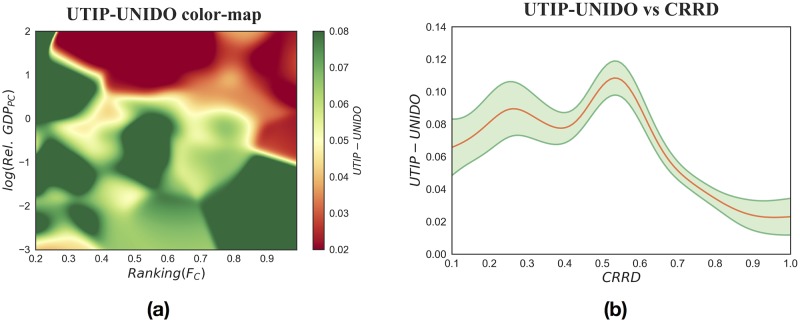
Pooling with the same features of Fig 3. (a): A tridimensional study of UTIP-UNIDO coefficient as a function of the Ranking of country Fitness and Relative GDP per capita. The color-map, obtained with a multivariate non-parametric kernel estimation, is a smoothed graphical representation of the UTIP-UNIDO coefficient for different values of the country Fitness and Relative GDP per capita. The diagonal variability of the color suggests that wage inequality between sectors, at this scale, is determined both by Relative GDP per capita and Fitness ranking and follows a pattern similar to the one predicted by Kuznets. (b): The relationship between *UTIP*—*UNIDO* coefficient and *CRRD* index. The red lines show a non-parametric kernel estimation and the green shadowed area represents a 90% confidence interval of *UTIP*—*UNIDO* expected values and have been computed with bootstrap. By employing the *CRRD* as an industrialization proxy, we recover the entire Kuznets curve not only its downward part.

Our starting point is the essentially declining relation between wage inequality and relative GDP per capita in Fig 3. Galbraith built the same plot, but used data from 1963 to 2008, and found the same downward sloping relation [27, 63]. He argued that this behavior was assimilable to the right part of the Kuznets curve, the only one that could had been detected, because during the investigated time interval much of the world’s countries had already experienced the first stages of industrialization [27, 63, 64]. However, by using only monetary information, it is easy to mistakenly asses the industrialization stage of a country. For instance, among the BRICS countries, despite similarities in GDP levels, there are heterogeneous endowments of capabilities. China shows a diversified and increasingly complex economy, while Russia is characterized by a fundamentally extractive economy, almost only based on the export of raw materials. This caused the divergent economic performances of the two countries in the last years [10]. When integrating the monetary information carried by GDP with that on country capabilities conveyed by Fitness we are able to grasp these crucial differences. And since here we are able to quantify development in a more detailed way, we can better tackle its relation to wage inequality. That is why, in Fig 4 we show the combined effect of Fitness and relative GDP on UTIP-UNIDO, at first separately in panel (a) and then simultaneously in panel (b). In panel (a) we show a wage inequality color-map obtained with a continuous non-parametric regression: from the diagonal variability of color we can see that both Fitness and GDP per capita play a role in the distribution of wages. This yields naturally to panel (b) where we represent development as a monodimensional variable by synthesizing the combined effects of Fitness and per capita GDP in the *CRRD*. In both the tridimensional and the monodimensional representation in the first stages of development there is an increase in wage inequality, while as development advances the relation becomes negative. Hence, our thesis is confirmed: by describing industrialization as a process which concerns both the complexity and the wealth of nations, it is possible to recover a pattern which corroborates Kuznets’ hypothesis and which is not clearly visible by selecting Fitness and GDP per capita separately.

Note that we find evidences of increasing wage inequality in the richest countries, the effect that Galbraith calls “Augmented Kuznets curve” [64], but it shows low significance and we do not enter into this debate since it is not relevant to our analysis.

To provide a more formal analysis of the results shown in Fig 4, we quantify the relation between industrialization and wage inequality with a parametric estimation. To tackle some essential features of the clearly non-linear relationship between development and wage inequality we will use squares of the variables. We are in no way arguing that this relationship is parabolic; however, the estimation of the best fitting parabola is fruitful because, on the one hand, it is a widely used technique since it allows for comparability with other studies and, on the other hand, it allows a quantitative measure of non-linearity.

In the following estimations the dependent variable is the natural logarithm of UTIP-UNIDO, while the regressors are different definitions of development: the ranking of GDP, Fitness and the *CRRD*, both in linear and quadratic terms. The results are presented in Tables 1 and 2 for different model specifications: Ordinary Least Squares and Fixed Effects Estimation respectively.

**Table 1:** Wage inequality: log(*UTIP*—*UNIDO*). OLS estimation, different model specifications.

Variable	(1)	(2)	(3)	(4)
Rank(*GDPpc*)^2^	-0.000135^⋆⋆⋆^ (0.000019)		-0.000192^⋆⋆⋆^ (0.000021)	
Rank(*GDPpc*)	0.0080^⋆⋆⋆^ (0.0030)		0.0221^⋆⋆⋆^ (0.0033)	
Rank(*Fitness*)^2^		-0.000060^⋆⋆⋆^ (0.000020)	0.000066^⋆⋆⋆^ (0.000022)	
Rank(*Fitness*)		-0.0025 (0.0036)	-0.0205^⋆⋆⋆^ (0.0039)	
*CRRD*^2^				-4.40^⋆⋆⋆^ (0.40)
*CRRD*				3.17^⋆⋆⋆^ (0.50)
*R*^2^	0.28	0.26	0.36	0.38

Standard errors in parentheses

Statistical Significance:

^⋆^ 10%,

^⋆⋆^ 5%,

^⋆⋆⋆^ 1%

**Table 2:** Wage inequality: log(*UTIP*—*UNIDO*). Fixed Effect estimation, different model specifications.

Variable	(1)	(2)	(3)	(4)
Rank(*GDPpc*)^2^	-0.000088^⋆⋆^ (0.000035)		-0.000076^⋆⋆^ (0.000035)	
Rank(*GDPpc*)	0.0084 (0.0056)		0.0067 (0.0056)	
Rank(*Fitness*)^2^		-0.000133^⋆⋆⋆^ (0.000037)	-0.000127^⋆⋆⋆^ (0.000037)	
Rank(*Fitness*)		0.0242^⋆⋆⋆^ (0.0064)	0.0228^⋆⋆⋆^ (0.0064)	
*CRRD*^2^				-3.63^⋆⋆⋆^ (0.96)
*CRRD*				3.7^⋆⋆⋆^ (1.1)
*R*^2^	0.012	0.015	0.025	0.016

Standard errors in parentheses

Statistical Significance:

^⋆^ 10%,

^⋆⋆^ 5%,

^⋆⋆⋆^ 1%

The tables show how, while both the ranking of GDP per capita (model 1) and the ranking of Fitness (model 2) present significant negative quadratic terms, the *CRRD* (model 4) outperforms both in terms of explained variance. And even combinations of the two variables and their squares (model 3) are not able to outperform the *CRRD* measure in a OLS framework. Since *CRRD* is a linear combination of the two rankings, this is a very strong result; it is even algebraically possible only because in model 3 we do not have the cross term Rank(*Fitness*)Rank(*GDPpc*). This shows that the *CRRD* allows to grasp a sizable amount of both Rank(*GDPpc*) and Rank(*Fitness*).

Notice that we are not considering any controls. Indeed, theoretically the Fitness measure already discounts any further possible dependency. Empirically, adding some possible dimensions of analysis (Education, Democracy, Freedom of Speech and Press, …) would be useful if we were to analyze the causal role of our *CRRD*. However, this is not the point of the exercise: as we said, it could well be that development and wage inequality co-evolve without direct causality. For this reason, by following Fig 4, it is not possible to infer if a change in the Fitness or GDP of a country provokes an increase or decrease in wage inequality.

We have completed the first part of our analysis: we were able to observe on a global scale a Kuznets-like curve connecting development, proxied by *CRRD*, and wage inequality. Although recovering the downward part of the inverted U-curve is already a solid result in the economic literature (see for example [25, 64–68]), we have retrieved the entire expected theoretical trajectory predicted by Kuznets.

### Within one country: The case of the United States

Here, we conduct an analysis that mirrors the global one, but we look in detail at the comparative development of the constituents of a single country, the counties of the United States. As the United States is one of the highest GDP countries, over the two analyzed decades it is placed in the rightmost part of a Kuznets curve, among developed and prosperous societies, and its expected inequality trend should be negative. Hence, if the underlying forces influencing the relation between wage inequality and development were the same among countries and within the United States, we would expect to look at a simple zooming of the rightmost part of the curve in Fig 4; however, if the relation were not scale-invariant and ended up being shaped differently, this would hint at scale-specific explanations of the development-inequality relationship.

As explained in Subsection *Sources of data*, for this analysis we focus on employment and wages regarding the approximately 3100 counties of the United States over the period 1990–2014. To do so, we employ the QCEW data-set in which industries are categorized according to the NAICS system. The maximum number of sectors for each county is *N*_*sectors*_ = 89, whereas the number of counties spans from $N_{counties}^{1990}=2700$ in 1990 to $N_{counties}^{2014}=3167$ in 2014. From this data, for each county and each year, we compute a Theil index that measures inequality in the distribution of NAICS sectoral wages—the Theil component defined in Eq 9—and, by implementing the method outlined in Subsection *Variables of interest*, we run the Fitness-Complexity algorithm as defined in Eq 5. We then evaluate the Complex Relative Rank Development index by employing county average wage instead of GDP per capita as monetary variable.

Counties constitute a variegated social context, in which the industrialization level, the predominant sectors, the dimension and ethnic composition of the population etc. can vary considerably. Per contra, in the United States a relatively uniform culture dominates and most of the economic policies are made at the state or at the federal level. By analyzing data regarding sub-units of a single national entity, we are able to control most of those institutional, economic and cultural factors that differ among countries. In fact, as shown by Moller et al., among counties cross-sectional institutional effects are non-significant, while they can have an influence over time [69].

**Fig 5:**
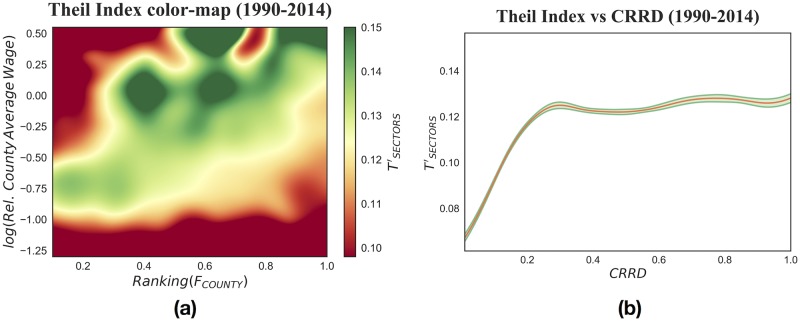
All years and counties are pooled over 1990–2014, for a number of counties spanning from 2700 to 3167, and 69663 total observations. (a): Theil index color-map, obtained with a multivariate non-parametric kernel estimation, a smoothed graphical representation of the variation of $T_{SECTORS}^{\prime }$. From the diagonal green band it is clear that the highest Fitness counties are the most unequal. (b): Between-sector Theil component versus Complex Relative Ranking Development index. We study the relationship with a non-parametric kernel regression: the red line depicts the kernel estimation of $T_{SECTORS}^{\prime }$ versus *CRRD* and the green shadowed area shows a 90% confidence interval of the expected value. The relationship is positive-sloping: as industrial development increases so does wage inequality, which then shows a plateau for high *F*_*C*_ values.

As in the previous section, we choose a continuous non parametric approach and pool all counties and sectors over 1990–2014. As shown in Fig 5, we see immediately that among sectors and within counties the development-wage inequality relationship does not follow a Kuznets curve. The curve is in fact upward sloping: the more complex and diversified the productive system of a county, the higher the wage inequality among sectors in it. The same behavior is uncovered in the wage inequality color-map, as can be seen from the diagonal green band in Fig 5 panel (a). This picture differs radically from multi-country analysis performed in the previous section. This difference in the cross-sectional relationship between development and wage inequality is the footprint of a difference in its determinants at different scales. The next section will be focused on what we can learn from this empirical difference.

## Behind the curve

The upturn in the relation of wage inequality to development that we found in the previous section puts into light a clear behavior change at different scales. Here we will sketch an analysis of the possible explanations of such lack of scale invariance.

Notice that we are not defining the direction of causality: we are only looking at correlations. Any causal relations between development and inequality will likely go in both directions, and even through omitted variables. It could be inequality that drives institutional change and development or vice versa, or, even, institutions could be the drivers of both inequality and development.

We are looking for the potential drivers of the relation that are decisive when comparing countries but are missing when examining wage inequality among the constituents of a country; the most obvious candidate for this are institutional factors. Therefore, consistently with Kuznets’ initial hypothesis, the latter can be good candidates in explaining the drop in wage inequality observed among countries and not among counties. Thus, we are now left with the determinants of the positive part of the relation of wage inequality to economic development observed for the United States. Kuznets followed a structural approach in explaining this inequality increase. Since within the United States institutions are not relevant, are then structural effects enough to determine the observed relation of wage inequality to economic development? How can changes in sectoral compositions along development lead to increased wage inequality? In our framework the most developed counties are often also the most diversified. By borrowing the terminology of biologists, industrial systems develop in a nested fashion [7, 8, 70]. This can be clearly noticed when observing the upper section of the county-sector matrix in Fig 4 in which almost all sectors are present in the highest Fitness counties (matrix rows), from the least to the most complex. A new sector is not introduced at random, but only when a productive system has developed the required basket of capabilities, and in this way gradually more and more complex sectors are introduced. For example, it would be unreasonable to believe that a region which has an economy that is strongly based on agriculture would abruptly start to produce electronic components for cars. This could only happen after a process of industrial, infrastructural, legislative and workforce skill adjustments.

**Fig 6:**
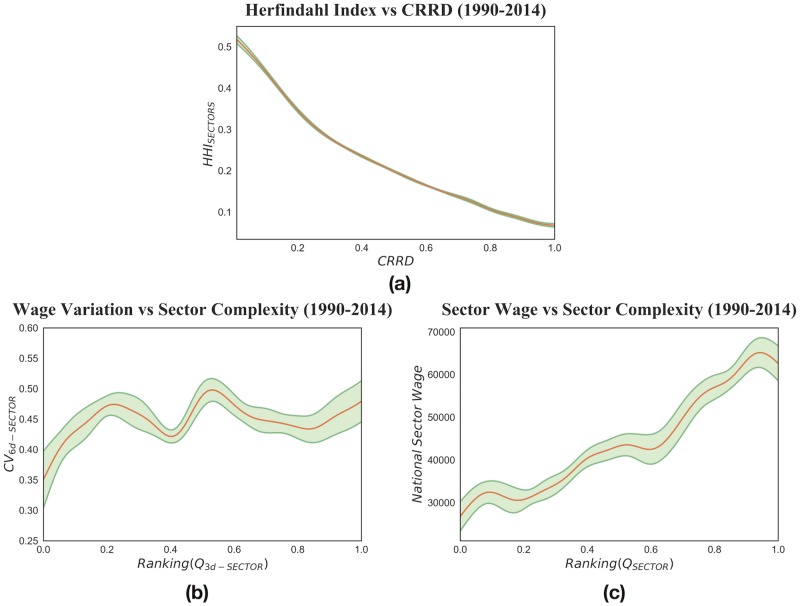
In this pooled analysis we relate the Herfindahl Index to the Complex Relative Ranking Development index, and the Coefficient of Variation of sectoral wages to Sector Complexity. (a) County Herfindahl Index versus County Complex Relative Ranking Development index. The Herfindahl Index is computed with the employment shares of 3-digit NAICS industries and, being a concentration measure, it shows that as industrial diversification increases so does wage inequality. (b) Sector Wage Coefficient of Variations versus Sector Complexity. The Coefficient of Variation is here computed by considering the variability of wages at 6-digit aggregation level within every 3-digit NAICS sector at which the complexity on the abscissa refers. The relation is approximately positive and, on average, in the most complex sector wages vary ∼15% more than in the least complex one. (c) Sector Wage versus Sector Complexity. National sectoral wages increase as the complexity level of the sector grows. Thus, not only average retributions rise sharply for growing complexity, but within complex sectors wage variability is also higher.

The nested structure of productive systems is strictly linked to wage inequality for three main reasons. Firstly, higher industrial diversification increases the space of possible jobs (independently from the order in which new sectors are introduced), and so intrinsically enlarges the inter-sectoral wage gap. Indeed, as can be seen in Fig 6 panel (a), we observe that the more developed the counties, the more diversified and unequal they are. In Fig 6 panel (a) we measure diversification with a Herfindahl-Hirschman Index (HHI) defined as such:

$$\begin{array}{c} HHI_{SECTORS}=\sum_{i=1}^{N}s_{i}^{2} \end{array}$$ (14)

where, for each county, *N* is the number of sectors and *s*_*i*_ is the workforce share of sector *i*. The Herfindahl-Hirschman Index ranges from 1/*N* to 1, because when all sectors have the same workforce shares *HHI*_*SECTORS*_ = 1/*N*, while when only one sector dominates *HHI*_*SECTORS*_ = 1.

Secondly, Fig 6 panel (c) displays that since the most complex sectors are more remunerated and by definition are present only in the most developed counties, inequality increases in the latter.

Thirdly, as can be observed in Fig 6 panel (b), another element in further enhancing this effect is that at the most disaggregated NAICS level, 6-digits, wages show higher variability in the most complex 3-digit sectors.

Therefore, the most developed counties, being the most diversified and in particular being the only ones that have the most complex sectors—which we showed are also the more internally unequal—show higher wage inequality.

So far our empirical findings are in agreement with the Kuznets’ narrative: in the first phases of development sectoral change drives wage inequality up, at both the observed geographical scales; in the second stage of development, instead, when comparing institutionally heterogeneous units, institutional change drives wage inequality down.

A natural continuation of this section will be analyzing the time evolution of the relationship by focusing both on a landscape in which institutions are predominant and on the United States in which the prevailing force is structural.

### Time evolution

We investigate whether the relationship between economic development and wage inequality both among countries and among counties remains intact or changes its features throughout the periods under consideration.

**Fig 7:**
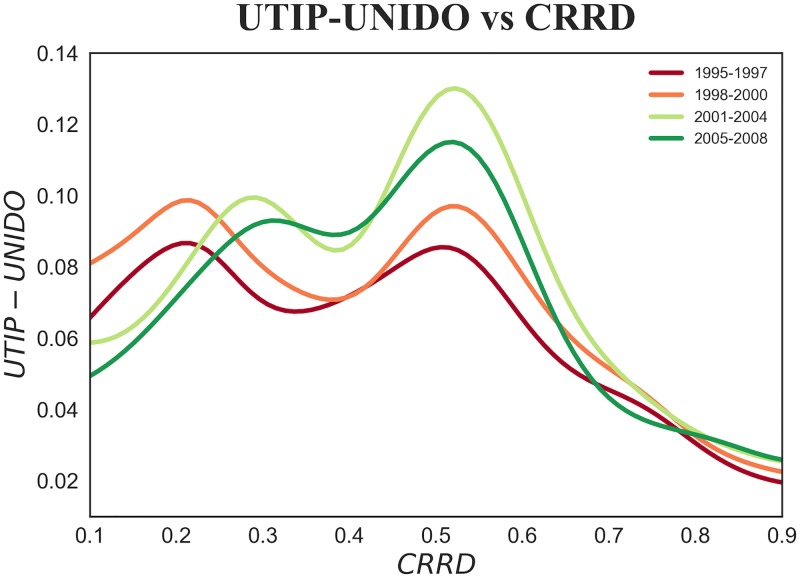
*UTIP*—*UNIDO* inequality measure versus *CRRD*. Pooling of countries and years for the four time intervals: 1995–1997, 1998–2000, 2001–2004 and 2005–2008. The colored lines show a non-parametric kernel estimation of UTIP-UNIDO expected values. The shape shown in Fig 4 panel (b) is preserved over the chosen time intervals.

Hence, as we did in the previous sections we divide our empirical analysis into two parts. Firstly, in the multi-country exercise we split the observation time 1995–2008 into four smaller time intervals. As can be observed in Fig 7, disregarding the global increase in inequality that lifts up the curve over time, the Kuznets-like functional form that came to light in the pooled analysis endures over time in the observed intervals.

**Fig 8:**
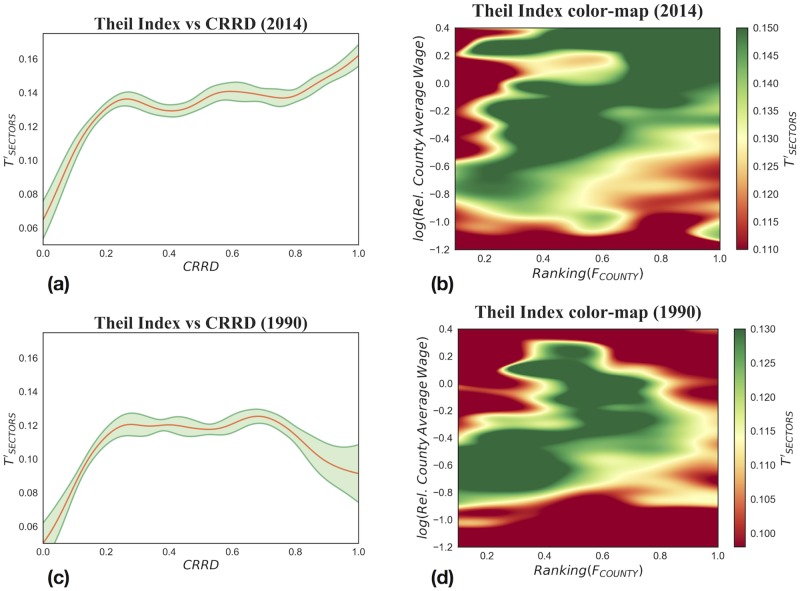
Relation between wage inequality and development at a county level, for 2014 in the top half and 1990 in the bottom half of the figure. Wage inequality is measured by the between-sector Theil component calculated from the distribution of sectoral wage at 3-digit NAICS aggregation level. The relations are analyzed with the same methods for both years. (a) and (c): $T_{SECTORS}^{\prime }$ versus *CRRD*. (b) and (d): Color-map of the variation of $T_{SECTORS}^{\prime }$ as a function of the Fitness and the Relative Average Wage of counties. In 2014, it is clear that as industrial development increases so does wage inequality. Differently from 2014, in 1990 counties’ wage inequality grows until a certain level of *CRRD* and then, after a plateau, starts to decrease.

**Fig 9:**
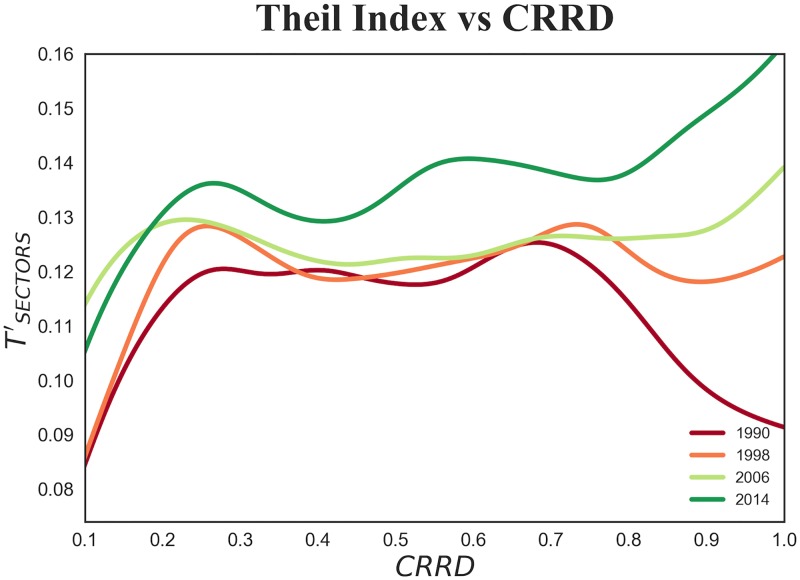
$T_{SECTORS}^{\prime }$ as a function of *CRRD* for US counties in the years 1990, 1998, 2006 and 2014. We observe two main features: (i) wage inequality increases over time; (ii) in 1990 we found a non monotonous behavior, while in the following years the second half of the curve experiences a turnaround, and as *CRRD* increases, inequality in the wage distribution among counties soars.

Secondly, in the analysis of the United States we study empirical data using the same approach for every year in the period under study and, for the sake of brevity, in Fig 8 we only display the observations for 1990 and 2014. In Fig 9 we show in a schematic way the time evolution of the Theil Index as a function of the Complex Relative Rank Development index. Notice that the longitudinally upswing in the United States’ wage inequality that has received much scholarly attention [32, 41] is clearly visible in Fig 9. In the early nineties the most developed counties show declining wage inequality. Later, there is a gradual trend reversal: the right tail of the curve starts to rise and, from the end of the nineties, the relation becomes monotonically increasing. This points to what we observed in the previous subsection: after the early nineties, more industrialized and developed counties tend to have high wage inequality.

**Fig 10:**
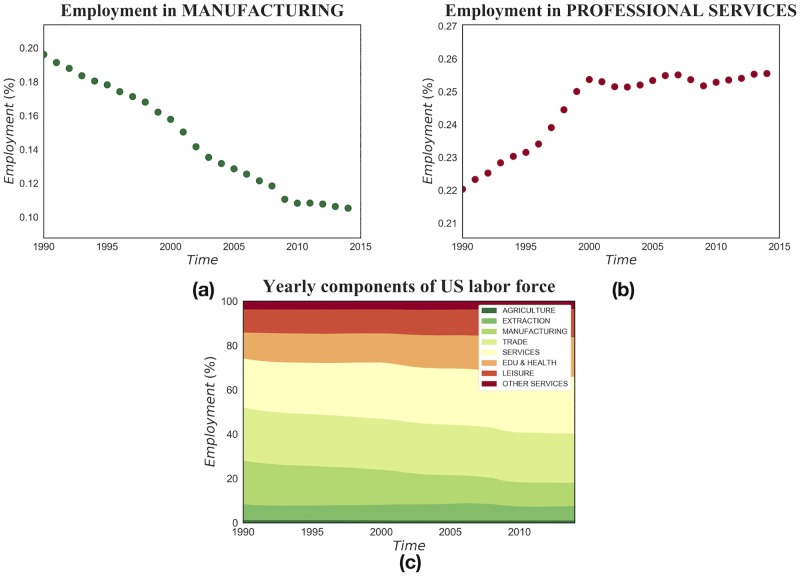
Evolution of sector employment. From (a) and (b) we can clearly observe the migration of labor force out of manufacturing (NAICS codes starting with 3) and the notable increase of employment in professional services (NAICS codes starting with 5).

These findings are consistent with the narrative that within the United States the wage inequality-development relation is driven by structural factors, while the declining part of the relation observed globally relies mainly on the institutional differences among countries. Indeed, despite the profound political shocks in the last decades, the role of institutions in the relation between development and wage inequality has not changed its core characteristics, independently from the direction of the causality arrow—whether economic inclusiveness is considered a driver for development or a reduction of inequality is seen as a consequence of development. On the contrary, when excluding institutional factors from the analysis, the role of the industrial structure of the United States has evolved over time. Indeed, in the last decades the United States and most OECD economies have experienced a radical inter-sectoral transition: the labor force has shifted massively from the historically more equal in term of pay, more unionized and regulated manufacturing sector, to the heavily deregulated and internally unequal service sector [71, 72] (we can appreciate the magnitude of the United States’ deindustrialization and consequent tertiarization by looking at the employment shares in Fig 10). This has increased overall inequality, but, more importantly for our analysis, it has crucially changed the form of the relation between inequality and development [43, 73]: a developed county in the early nineties was relatively equal in term of pay as its economy was based on manufacturing, while in more recent years it has become a service-intensive county in which employment is rising both in high-paying services and in low-paying services, where the majority of the workforce has been increasingly concentrated.

Put in other words, the wage inequality-development relation, within the United States, still has its roots in Kuznets’ hypothesis: a consistent pattern of inequality as a county develops exists and is caused by the movement of the labor force among sectors. What changed, driven by trade and technology shifts, is the direction of employment flows among sectors along development.

**Fig 11:**
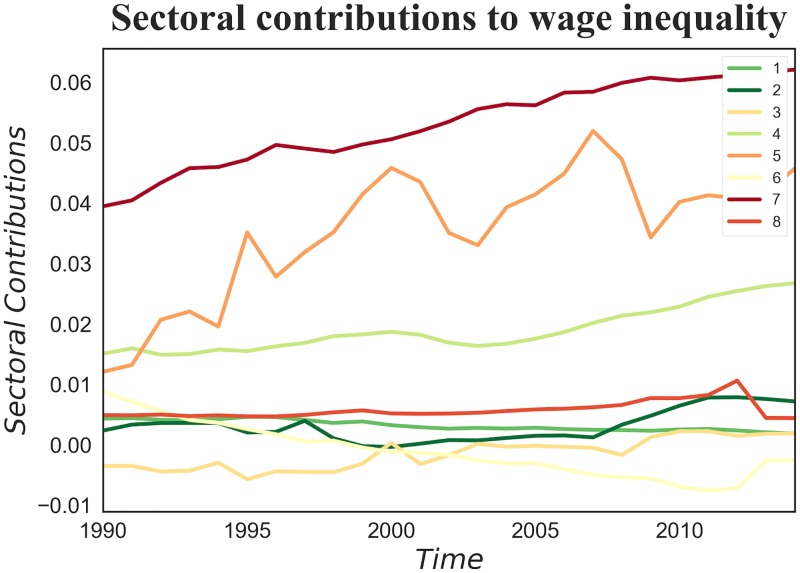
The macro-sectoral contributions to wage inequality computed with the Shapley value over the time interval 1990–2014. Where the macro-sectors depicted in the figure are: 1 = agriculture, 2 = extraction, 3 = manufacturing, 4 = trade, 5 = professional services, 6 = education and health services, 7 = leisure industry, 8 = other services. The major effect on inequality is given by the service industries.

Can we directly connect the change, from 1990 to 2014, in the shape of the relation between *CRRD* and wage inequality among counties, shown in Fig 9, to this deindustrialization process? While a clear empirical answer to this question is not possible, we can offer a further empirical clue by looking at the marginal contributions of each industrial macro-sector to overall wage inequality. These contributions are computed with the Shapley Value, a technique used in cooperative game theory [74]; the details of this procedure are provided in the S2 Appendix. The general idea is to decompose wage inequality among sectors by looking at what wage inequality would be if we removed from the economy one or more sectors at a time. Fig 11 illustrates not only that the service industries (in particular those with NAICS codes starting with 4, 5 and 7) contribute majorly to between sector wage inequality, but more importantly it shows how the increase of inequality, a surge from 0.8 to 1.5 from 1990 to 2014, is *only* due to those same industries: excluding services, both in 1990 and 2014, the contributions to inequality of all the other sectors sum up to 0.2.

## Conclusion

In this paper we have addressed the relationship between wage inequality and development. An important feature of this work is the proper quantitative identification of the process of development by using two novel applications of complex system analysis. Firstly, we have generalized the Fitness-Complexity metric: by weighting the importance of a certain sector in a region by its wage volume distribution compared to a reference distribution, in the evaluation of the complexity of an economy we have considered industrial sectors and generic geographic areas. This generalization to labor data instead of export data allows us, on the one hand, to take into account also those sectors that only have a role in domestic markets, and, on the other hand, to compute the Fitness of sub-country geographical entities, making possible to extend the economic complexity analysis at different scales. Secondly, by combining Fitness and a monetary value—in turn GDP per capita and county average wage—we are able to define the Comparative Relative Rank Development index, a measure of economic performances that captures both monetary elements and information on the complexity and so forth on the underlying capabilities of an economic system.

Once we have defined our development measure, in order to investigate the features of the cross-sectional relation that links the distribution of labor income to the process of industrialization and development, we have conducted an analysis *à la* Kuznets. Firstly, we have focused on a between-countries scale and, pooling data over the time interval 1963–2008, we have uncovered the expected Kuznets-like pattern. The curve, that appears persistent longitudinally, was not visible putting wage inequality in relation with a more classic measure of development such as relative GDP per capita. With the purpose of checking if such curve keeps the same functional form when observing the constituents of a single country, we have then looked for the same relationship in a politically homogeneous environment: the counties of the United States over the time window 1990–2014. The Kuznets curve that appeared in the multi-country analysis is not recovered within the United States, there is in fact a substantial trend reversal: at a county level wage inequality increases with growing development stages. Furthermore, the functional form of the relation varies over time: in 1990 it traces an inverted parabola curve, while in the following years a monotonically increasing behavior emerges.

Our findings are coherent with a Kuznets’ process in which, in the first stage of development, the predominant force driving the wage inequality-development relation is structural change—the shift in the labor force composition that lifts inequality up with development at both our observation scales. With growing development and diversification, workers have access to more options, with more heterogeneous wages; this is reflected in the positive part of the inequality-development relation. In the second stage of this process, when considering politically heterogeneous environments, institutional change bends the curve down reducing wage inequality. We have highlighted that our empirical facts hint to such a narrative for two main reasons. By comparing the empirical relationship between development and wage inequality at different geographical scales, we have observed how the declining part of the curve disappears among the politically homogeneous US counties. Moreover we have shown that while globally the relation between development and wage inequality has constant features over time, in the US counties both the sectoral composition and the form of the relation have drastically changed.

## Supporting information

S1 Appendix Robustness check: A Gini Coefficient of wage inequality between sectors. In this appendix we test the robustness of our results and show that they are unaltered when measuring wage inequality among sectors within countries/counties with a Gini coefficient instead of a Theil index.
(PDF)

S2 Appendix Shapley values. In this appendix we describe the procedure used to compute the Shapley values that quantify the sectoral contributions to overall wage inequality that are shown in Fig 11.
(PDF)

S3 Appendix Colorblind friendly figures. In this appendix we reproduce all the figures of the paper in a palette more accessible to red-green blind people.
(PDF)
