# Supplementary material for: Economic development and wage inequality: A complex system analysis
Source: PLoS One. 2017 Sep 19;12(9):e0182774. doi: 10.1371/journal.pone.0182774 (PMC5604954; doi:10.1371/journal.pone.0182774)
Supplement: S1 Appendix — In this appendix we test the robustness of our results and show that they are unaltered when measuring wage inequality among sectors within countries/counties with a Gini coefficient instead of a Theil index. (PDF) [file pone.0182774.s001.pdf]

Economic Development and Wage Inequality: a Complex System Analysis –S1 Appendix

Angelica Sbardella[1,3], Emanuele Pugliese[2], Luciano Pietronero[1,2]

1 ISC-CNR – Institute of Complex Systems, Rome, Italy
2 Department of Physics, Sapienza Università di Roma, Rome, Italy
3 Faculty of Economics, Università degli Studi di Roma Tor Vergata, Rome, Italy

Robustness Check: a Gini Coefficient of Wage Inequality Among Sectors

In order to study wage inequality within US counties among sectors, we chose a between group Theil index, as defined in Section *Variables of interest.* These entropy-based inequality measures are decomposable into a within group component and a between group component, however they are not immune to single outliers [1]. With the aim of testing the robustness of our results, in this Appendix we delineate the same analysis scheme of Section *Within one country: the case of the United States*, however to measure wage inequality we use a Gini coefficient between-sector. The latter is an inequality measure which have been proved to be stabler to single outlying observations at the top or at the bottom of the distribution, and is the golden standard of inequality measures in the economic literature.

For a population of n individuals and a discrete income distribution $\mathbf{x} \in \mathbb{R}_+^n$ with $x_p \leq x_{p+1} \,|\, p = 1, \cdots, n$, the Gini coefficient independent from the population is defined as follows:

$$\tilde{G}(\mathbf{x}) = \frac{2}{n \sum\limits_{p=1}^{n} x_p} \left(\sum_{p=1}^{n} p\, x_p \right) - \frac{n+1}{n}. \tag{1}$$

We consider a partition of the working population in N_s groups, where N_s is the number of industrial sectors present in the society under study. If P is the total number of workers, y_i is the average wage and p_i is the number of workers of the i-th sector, we consider a vector of average wages $\mathbf{y} \in \mathbb{R}_+^{N_s}$ arranged in ascending order from the worst $(i = 1)$ to the best paid job $(i = N_s)$. For this $\mathbf{y}$ vector with $y_i < y_{i+1} \;|\, i = 1, \cdots, N_s$ and for a population of P workers, we obtain a Gini coefficient of this kind:

$$G_{SECTORS} = \frac{2 \sum\limits_{i=1}^{N_s} a_i\, y_i}{P \sum\limits_{i=1}^{N_s} p_i\, y_i} - \frac{P+1}{P}, \quad y_i \leq y_{i+1} \tag{2}$$

where, for the i-th sector:

$$b_i = \sum_{j=1}^{i} p_j;$$

$$a_i = \frac{(b_i - b_{i-1}) \cdot (b_i + b_{i-1} + 1)}{2}, \quad \text{for } i > 1;$$

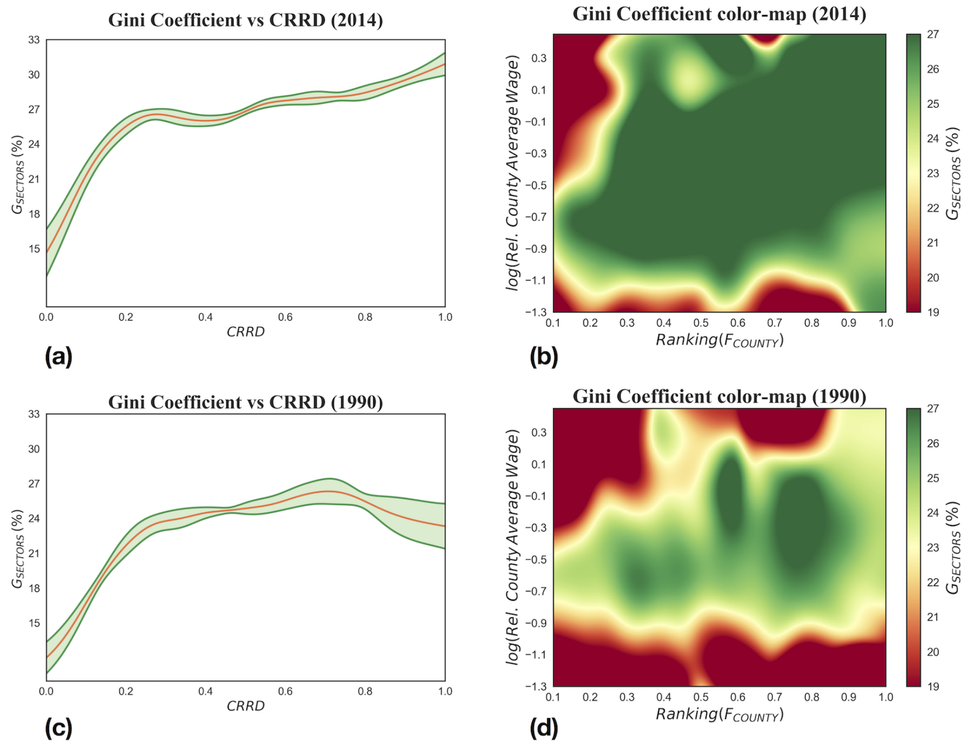

Figure S1 1. (a) and (c): $G_{SECTORS}$ versus $CRRD$ respectively in 2014 and 1990. (b) and (d): Color-map of the variation of $G_{SECTORS}$ as a function of F_{COUNTY} and relative Average Wage of counties respectively in 2014 and 1990. The relationships found by measuring wage inequality with a between-sector Theil component are in agreement with the results shown in this figure. In fact, in 1990 the movement of wage inequality recalls a Kuznets curve, while in 2014 it increases monotonically with the advance of industrialization. This is confirmed by the Spearman correlations between the $G_{SECTORS}$ and $T'_{SECTORS}$ which are, respectively for 1990 and 2014, $r_{1990}(G_{SECTORS}, T'_{SECTORS}) = 0.97$ and $r_{2014}(G_{SECTORS}, T'_{SECTORS}) = 0.98$.

$$a_1 = \sum_{j=1}^{p_1} j, \quad \text{for } i = 1.$$

As it shown in Fig 1, when we measure inequality with such a Gini coefficient the relation of wage inequality with development is concordant to that found with the between sector Theil component: in 1990 wage inequality follows a Kuznets-like pattern, while in 2014 is monotonically increasing. These qualitative findings are reinforced by the high values of the Spearman correlations between the two inequality indexes for 1990 and 2014:

$$\begin{aligned} r_{1990}(G_{SECTORS}, T'_{SECTORS}) &= 0.97 \\ r_{2014}(G_{SECTORS}, T'_{SECTORS}) &= 0.98. \end{aligned}$$

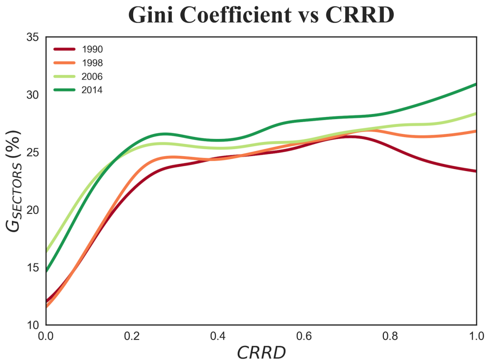

Figure S1 2. $G_{SECTORS}$ as a function of $CRRD$ for US counties in 1990, 1998, 2006 and 2014. Here, again, the patterns are concordant to those found with $T'_{SECTORS}$.

Moreover, as shown in Fig 2, the time evolution of the Gini coefficient versus the Complex Relative Rank Development index is also concordant to the one with the Theil measure. Thus, we can conclude that the results obtained employing a Theil measure for the analysis of wage inequality in US counties are solid to the use of other inequality metrics.

References

1. Cowell FA, Victoria-Feser MP. Robustness properties of inequality measures. Econometrica: Journal of the Econometric Society. 1996; 64: 77–101.
