# Supplementary material for: Economic development and wage inequality: A complex system analysis
Source: PLoS One. 2017 Sep 19;12(9):e0182774. doi: 10.1371/journal.pone.0182774 (PMC5604954; doi:10.1371/journal.pone.0182774)
Supplement: S2 Appendix — In this appendix we describe the procedure used to compute the Shapley values that quantify the sectoral contributions to overall wage inequality that are shown in Fig 11. (PDF) [file pone.0182774.s002.pdf]

Economic Development and Wage Inequality: a Complex System Analysis –S2 Appendix

Angelica Sbardella[1,3], Emanuele Pugliese[2], Luciano Pietronero[1,2]

1 ISC-CNR – Institute of Complex Systems, Rome, Italy
2 Department of Physics, Sapienza Università di Roma, Rome, Italy
3 Faculty of Economics, Università degli Studi di Roma Tor Vergata, Rome, Italy

Shapley Values

In cooperative game theory, the Shapley value has been introduced as a scheme of distribution of the total gains among players with a coalition-dependent contribution to the total gain [1]. The rationale behind the scheme is that the Shapley value is the only distribution such that the total gain is always fully distributed among players. Additionally, such distribution is linear (if the total gain doubles, each player gets twice the share), symmetric (two players with equal contributions in every possible coalition get the same share) and fulfills the "Zero player" condition (a player not having a contribution in any coalition will get a share equal to zero).

This technique has recently been used to decompose other values in different contributions when the same properties are required. For example, Huettern shows that the Shapley value is the only way to decompose the R^2 of a multi-dimensional regression between the single regressors, if the decomposition is required to have properties formally equivalent to the ones stated before for cooperative games [2]. In our case, in Section *Time evolution* we use the same procedure to decompose the total inequality of a county among industrial sectors, according to same index I of inequality. To illustrate the procedure to compute the Shapley values, let us start with an empty economy and compute the inequality index at each step while adding all the k industries in a given order P. The Shapley value for a particular industry j is computed as the average marginal contribution to the inequality index of adding the industry j to the economy over all $k!$ possible permutations $P \cup K$ of the k industries.

Since the marginal contribution of industry j does not depend on the order of both the industries introduced before and after, by defining P_i as the set of industries preceding i in the permutation P, the share of the inequality index assigned to industry j can be written as:

$$I_j = \frac{1}{k!} \sum_{P \subseteq K} I(P_j \cup \{x_j\}) - I(P_j).$$

This formula describes the sectoral contributions to wage inequality represented in Fig 1.

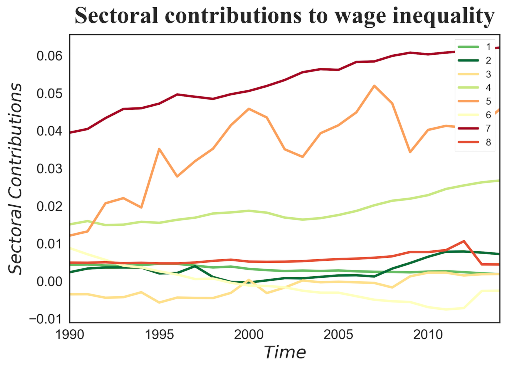

Figure S2 1. The macro-sectoral contributions to wage inequality computed with the Shapley value over the time interval 1990–2014.

References

1. Shapley LS. A value for n-person games. Contribution to the Theory of Games. 1953; II: 307–317.

2. Huettner, F and Sunder, M Axiomatic arguments for decomposing goodness of fit according to Shapley and Owen values. Electronic Journal of Statistics. 2012; 6: 1239–1250.
