# Supplementary material for: Economic development and wage inequality: A complex system analysis
Source: PLoS One. 2017 Sep 19;12(9):e0182774. doi: 10.1371/journal.pone.0182774 (PMC5604954; doi:10.1371/journal.pone.0182774)
Supplement: S3 Appendix — In this appendix we reproduce all the figures of the paper in a palette more accessible to red-green blind people. (PDF) [file pone.0182774.s003.pdf]

Economic development and wage inequality: a Complex System Analysis –S3 Appendix

Angelica Sbardella[1,3], Emanuele Pugliese[2], Luciano Pietronero[1,2]

1 ISC-CNR – Institute of Complex Systems, Rome, Italy
2 Department of Physics, Sapienza Università di Roma, Rome, Italy
3 Faculty of Economics, Università degli Studi di Roma Tor Vergata, Rome, Italy

Color-blind Friendly Figures

The chromatic choice of the paper, which plays with a red-green contrast, is thought to visualize at a first glance the contrast between high (red) and low (green) inequality. However, we are aware that a red-green palette may undermine the possibility of decoding exactly the information displayed in the figures. This Appendix offers a more accessible palette, in which all the figures of the paper and S1 Appendix are reproduced. The figure numbering is the same of the paper and S1 Appendix; captions can be easily understood by reading blue instead of green.

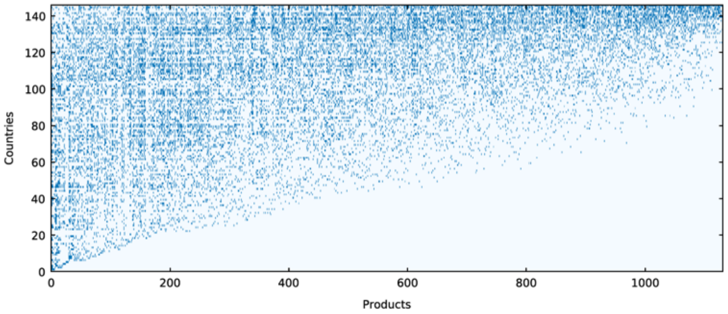

Figure S3 1. Fig 1 of the paper in a red-blue palette.

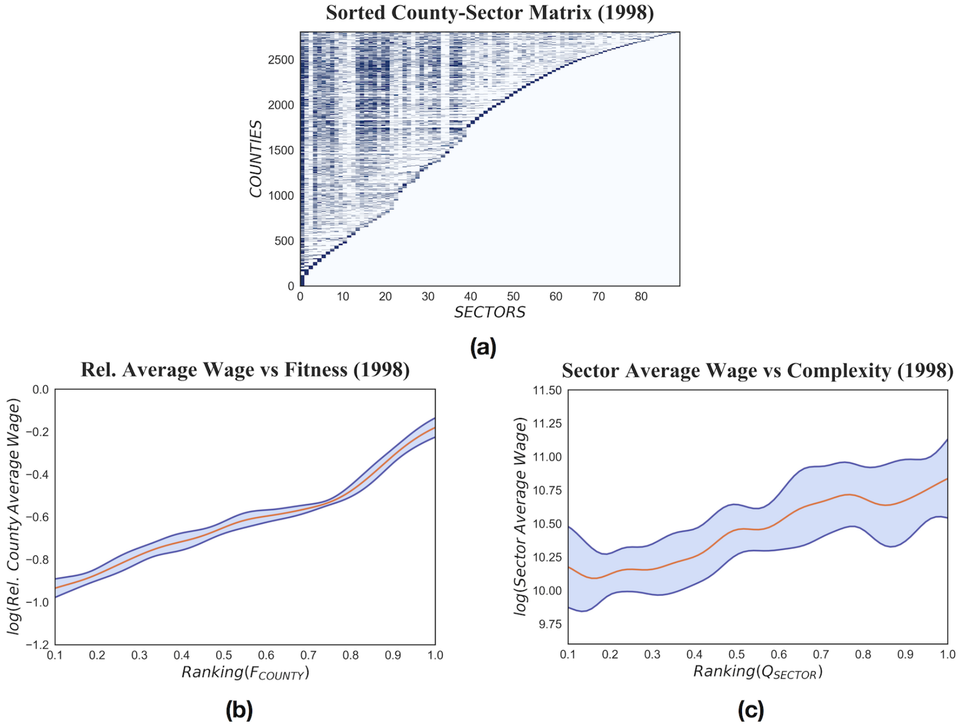

Figure S3 2. Fig 2 of the paper in a red-blue palette.

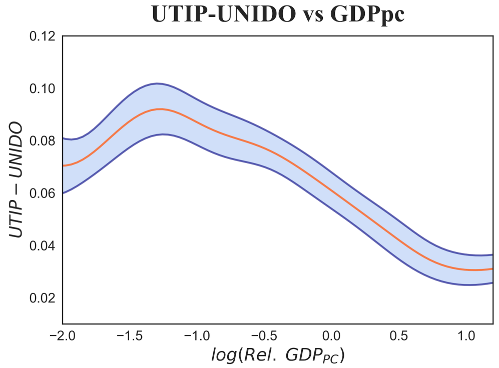

Figure S3 3. Fig 3 of the paper in a red-blue palette.

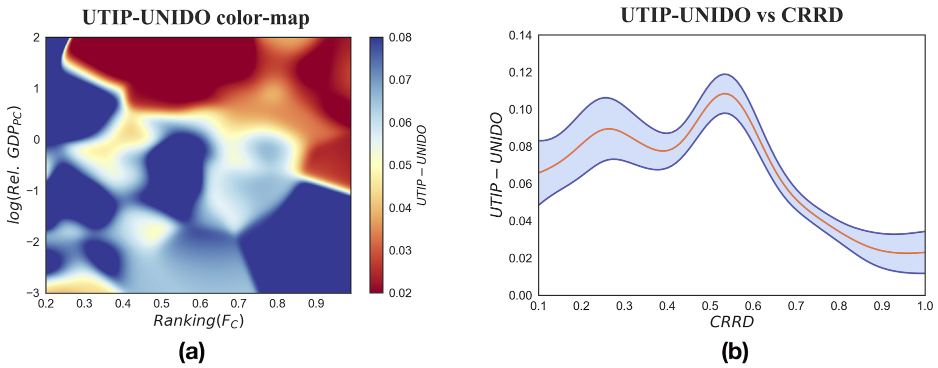

Figure S3 4. Fig 4 of the paper in a red-blue palette.

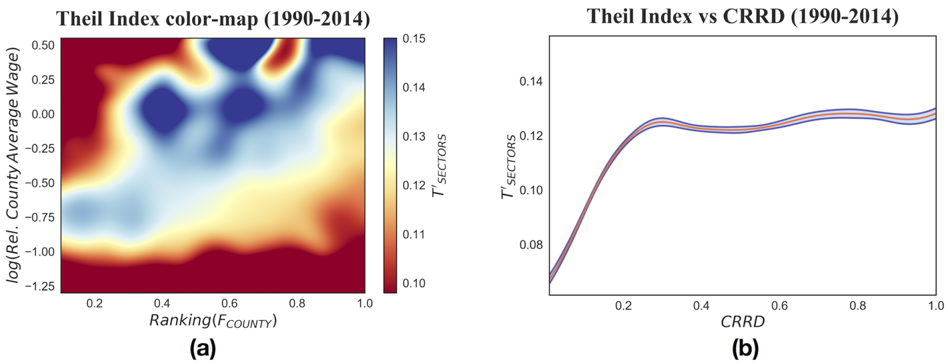

Figure S3 5. Fig 5 of the paper in a red-blue palette.

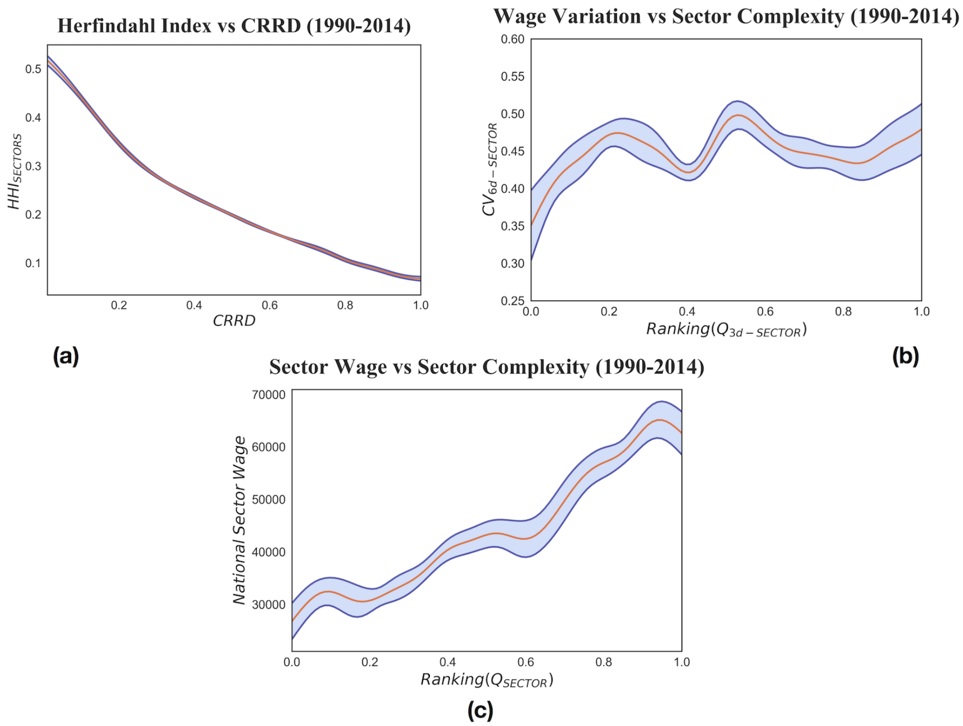

Figure S3 6. Fig 6 of the paper in a red-blue palette.

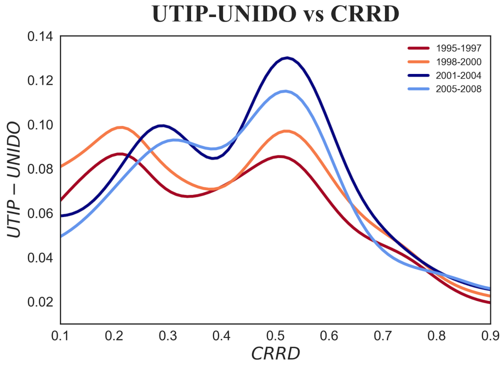

Figure S3 7. Fig 7 of the paper in a red-blue palette.

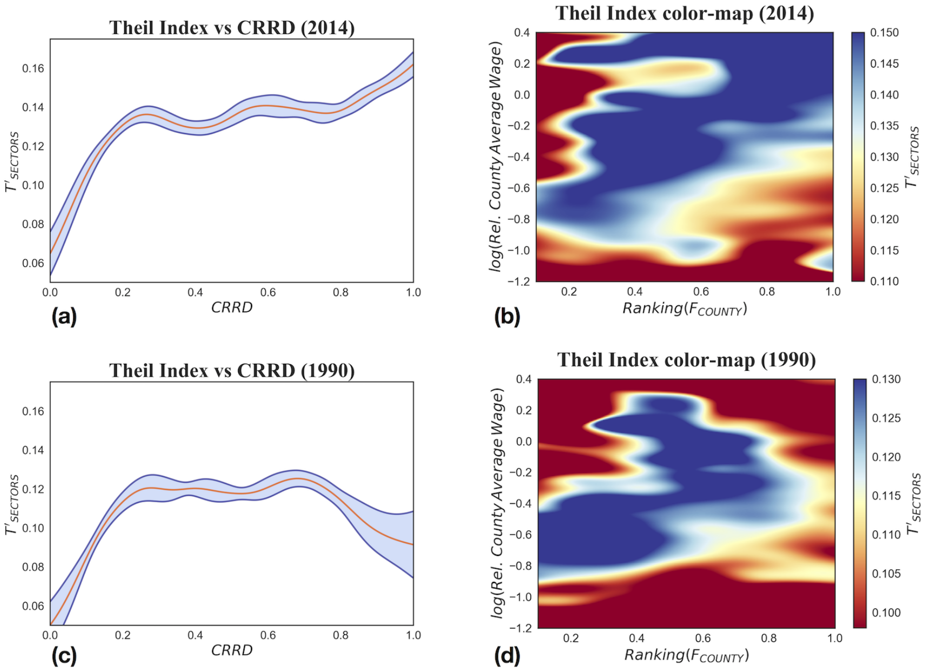

Figure S3 8. Fig 8 of the paper in a red-blue palette.

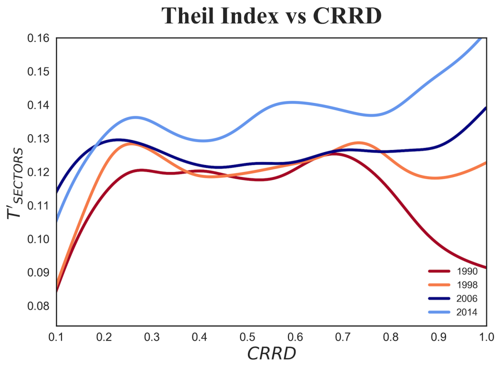

Figure S3 9. Fig 9 of the paper in a red-blue palette.

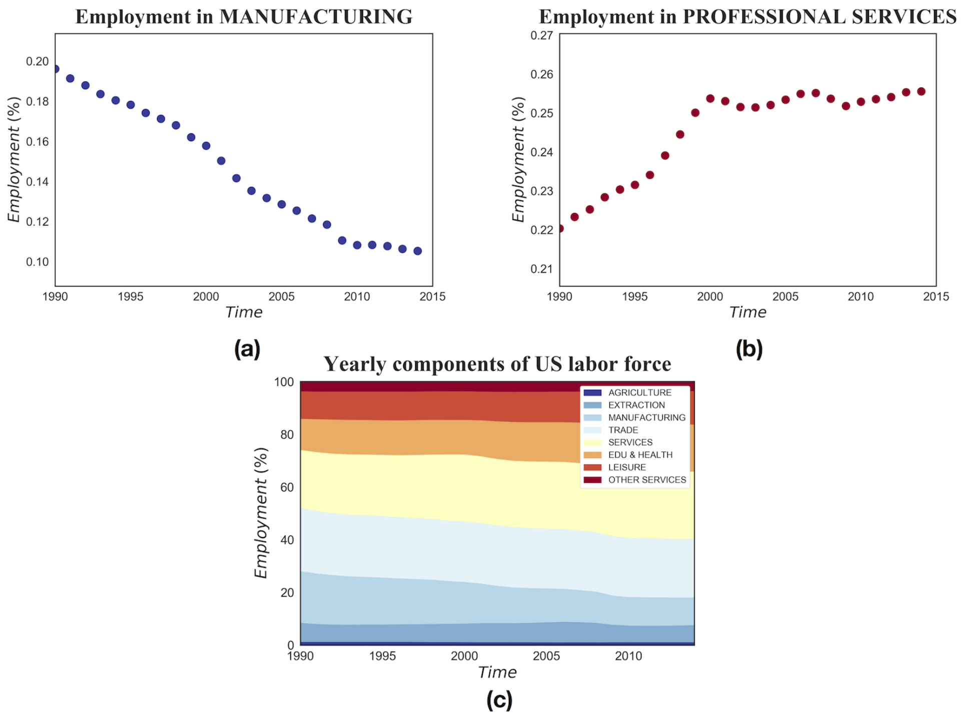

Figure S3 10. Fig 10 of the paper in a red-blue palette.

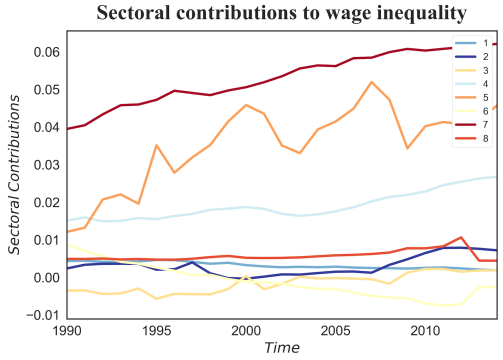

Figure S3 11. Fig 11 of the paper in a red-blue palette.

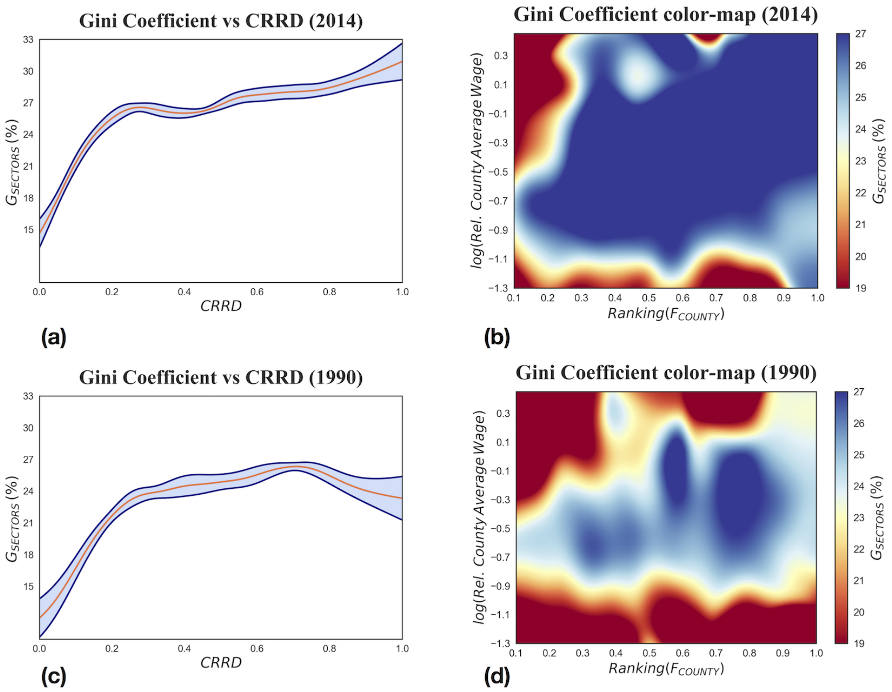

Figure S3 1. Figure S1 1 of S1 Appendix in a red-blue palette.

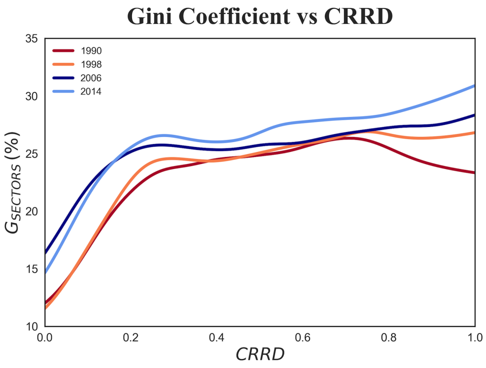

Figure S3 2. Figure S1 2 of S1 Appendix in a red-blue palette.
